# The non-ecology of the rich: elite power and the politics of ecological inequality

**DOI:** 10.3389/fsoc.2026.1986552

**Published:** 2026-10-30

**Authors:** Sonia Paone

**Affiliations:** Department of Political Sciences, University of Pisa, Pisa, Italy

**Keywords:** Bourdieu, ecological limits, elites, environmental privilege, political ecology, socioecological inequality, sustainability

## Abstract

This integrative conceptual analysis develops the notion of the non-ecology of the rich to examine how structurally privileged positions shape relations to ecological crisis. Bringing political ecology into dialogue with Bourdieusian social theory, it argues that ecological inequality concerns not only unequal environmental impacts and exposure, but also unequal capacities to negotiate ecological constraints, displace their costs, and define legitimate responses to them. Building on research on carbon inequality, environmental privilege, ecological habitus, luxury and sustainable consumption, environmental gentrification, and green capitalism, the article conceptualizes the non-ecology of the rich through three interrelated dimensions: insulation from ecological constraints, externalization of ecological costs, and symbolic authority over legitimate sustainability. Its analytical contribution lies not in identifying previously unknown mechanisms or a distinct set of elite environmental practices, but in integrating these dimensions within a relational framework that connects material, spatial, and symbolic forms of socioecological power. The article reconsiders the relationship between the environmentalism of the poor and the non-ecology of the rich as a structural asymmetry rather than a categorical opposition between homogeneous social groups. Drawing on existing research on mobility, environmentally privileged urban space, and private adaptation, it explores how different combinations of insulation, externalization, and symbolic authority may become visible across socioecological contexts. The concept ultimately shifts attention from what affluent actors do to the environment toward the unequal social relations through which ecological limits are experienced, distributed, negotiated, and politically defined. In doing so, the article proposes a conceptual framework for investigating ecological inequality as a configuration of material, spatial, and symbolic power.

## 1 Introduction

Ecological crisis is frequently articulated through a universalizing language: the planet is in danger, humanity must act, and responsibility is shared. This language reflects the planetary scale of ecological disruption, but it can also obscure the profoundly unequal social relations through which environmental crises are produced, experienced, and governed. Political ecology and environmental justice scholarship have long demonstrated that exposure to environmental harm, responsibility for ecological degradation, and the capacity to respond to it are unevenly distributed across class, racial, geographical, and political divisions (Agyeman et al., 2003; Escobar, 1996; Pulido, 2000; Pellow, 2007). There is, in this sense, no socially undifferentiated ecological “we.”

Much of this scholarship has rightly focused on populations disproportionately exposed to extraction, pollution, displacement, resource scarcity, and infrastructural abandonment. Yet ecological inequality also has another dimension. Social groups differ not only in their exposure to environmental harm or contribution to ecological degradation, but also in their capacity to create distance from ecological constraints, to displace some of their costs, and to influence the terms through which legitimate ecological responses are defined. The distribution of ecological vulnerability is therefore inseparable from the distribution of protection, mobility, resources, and authority.

This article examines this second dimension through the concept of the non-ecology of the rich. The concept asks a relational question: how does the concentration of social resources alter the terms on which ecological limits are encountered? Rather than treating affluent environmental practices primarily as expressions of hypocrisy, inconsistency, or individual lifestyle, I argue that they must be situated within unequal socioecological relations. Economic, cultural, social, and symbolic resources can shape not only what actors consume or how they respond to environmental concerns, but also their capacity to mediate exposure, access protection, relocate costs, and participate in defining legitimate sustainability.

The prefix “non” does not denote an absence of ecological relations or a simple rejection of environmental concern. It identifies a structurally produced capacity to suspend, mediate, or relocate ecological constraint, and hence to experience particular limits as negotiable rather than immediately binding. The negation is therefore simultaneously structural and phenomenological: it is grounded in unequal resources and expressed in a socially differentiated experience of ecological limits.

To develop this argument, I bring political ecology into dialogue with Bourdieusian social theory. I do not posit a unified global “ecological field” in the strict Bourdieusian sense. Ecological governance and environmental practice unfold across partially overlapping urban, national, transnational, corporate, infrastructural, and everyday arenas whose boundaries and forms of autonomy vary considerably (Buchholz, 2016; Jong, 2024). I therefore use the term socioecological space to designate a relational and multi-scalar configuration in which actors occupy unequal positions according to the resources they can mobilize and in which struggles concern both material conditions and their legitimate classification.

The analytical problem is therefore not simply why affluent groups may display environmentally contradictory behaviour. It is how privileged social positions can reorganize the relation to ecological constraint itself. What resources make it possible to negotiate or defer particular limits? Through which relations are ecological costs displaced elsewhere? And how can material and cultural advantage be converted into authority over the legitimate meaning of sustainability? These questions provide the point of departure for the concept developed in this article.

Existing scholarship provides several important points of departure for this argument. Research on carbon inequality has established the disproportionate contribution of high-income groups to carbon emissions (Chancel, 2022), while work on luxury consumption and affluent carbon footprints has connected these impacts to consumption patterns, investment, and the broader economic and political power of wealthy actors (Kenner, 2019). These approaches make visible the unequal contribution of social groups to ecological degradation, but ecological inequality also concerns the unequal capacity to avoid, mitigate, or displace its consequences.

The literature on environmental privilege extends the analysis in precisely this direction, showing how economic, political, and cultural power produces unequal access to environmental goods, amenities, and protection from environmental harm (Park and Pellow, 2011; Pellow and Nyseth Brehm, 2013). Research on environmental and green gentrification further demonstrates the spatial mechanisms through which environmental improvement can generate exclusion and reproduce classed geographies of privilege (Checker, 2011; Gould and Lewis, 2017). Environmental inequality, from this perspective, concerns not only unequal ecological impacts but also unequal access to protection, environmental quality, and resilient spaces.

A further body of scholarship shifts attention from access and exposure to practices, dispositions, and legitimacy. Work on ecological habitus and sustainable consumption has shown that environmental dispositions are socially differentiated and that practices coded as “green” can function as markers of distinction and moral worth (Haluza-DeLay, 2008; Carfagna et al., 2014; Anantharaman, 2022). At a different scale, critiques of green capitalism have demonstrated how environmental concern can be incorporated into processes of accumulation and legitimation rather than necessarily disrupting them (Prudham, 2009). Taken together, these literatures establish that ecological inequality involves differentiated impacts, unequal protection, socially structured environmental practices, and struggles over the legitimacy of ecological responses.

A contrasting strand of scholarship, associated with ecological modernization, places greater emphasis on the capacity of technological innovation, institutional reform, and market mechanisms to reconcile economic development with environmental improvement (Mol et al., 2009). The present framework does not deny that such reforms can reduce ecological pressures. It asks, instead, how their benefits, costs, and conditions of access are distributed, and whether apparently general solutions depend upon unequal capacities for protection and externalisation.

The non-ecology of the rich builds on these insights rather than proposing an alternative vocabulary for phenomena already identified. Its analytical specificity lies in bringing together three capacities that adjacent literatures tend to foreground separately: relative insulation from ecological constraints, externalisation of ecological costs, and symbolic authority over legitimate sustainability. Concentrations of capital may enable actors to distance themselves from particular constraints, displace costs across populations and territories, and convert material and cultural advantage into authority over ecological responsibility. The concept thus contributes to the sociology of environmental stratification by specifying how unequal impacts and protection can become connected to the unequal power to negotiate and define ecological limits.

The distinction can be stated more precisely. Carbon inequality and luxury-emissions research quantify the disproportionate ecological impact of high-income groups; environmental privilege and climate-apartheid approaches identify unequal access to protection and the socio-spatial segregation of environmental security (Rice et al., 2022); ecological unequal exchange, the imperial mode of living, and the externalization society trace how affluent ways of life depend on transfers of materials, labour, and harm across core-periphery relations (Givens et al., 2019; Lessenich, 2019; Brand and Wissen, 2021). Work on ecological capital, meanwhile, draws attention to environmental assets, competences, and distinctions that can be accumulated or converted into advantage. The non-ecology of the rich does not supersede these approaches. It addresses the specific analytical problem of how protection from constraint, displacement of cost, and authority to classify responses as sustainable may be joined within the same relational configuration. Its originality is therefore synthetic and explanatory: the individual mechanisms are established, but their conversion into one another has rarely been treated as the object of a single framework.

The concept shifts the analytical question from what the rich do to the environment to the structurally differentiated relations through which affluent actors encounter ecological limits, their costs, and their political definition. High-carbon consumption, environmental privilege, green distinction, protected urban environments, and private adaptation are not interchangeable phenomena; they are sites in which different elements of this positional logic may become visible. The non-ecology of the rich names neither a lifestyle nor a fixed set of practices, but a relational configuration of power within an unequal socioecological space.

The terms “rich” and “poor” consequently designate relational positions rather than homogeneous groups endowed with fixed ecological dispositions. Affluent actors can experience vulnerability and develop ecological commitments that cannot be reduced to symbolic distinction; less privileged populations are neither uniformly territorially dependent nor motivated only by necessity. The environmentalism of the poor and the non-ecology of the rich should therefore be read as different analytical perspectives on a structural asymmetry, not as a binary opposition between necessity and choice, authentic and symbolic environmentalism, or vulnerability and freedom.

### 1.1 Conceptual approach

The article uses a problem-driven integrative conceptual analysis rather than a systematic review. The literatures were selected because they illuminate different aspects of a common question: how privileged social positions alter the encounter with ecological limits. Research on carbon inequality and ecologically unequal exchange was used to identify unequal impacts and cost displacement; work on environmental privilege, green gentrification, and adaptation to identify differentiated protection; and scholarship on ecological habitus, sustainable consumption, green capitalism, and Bourdieusian symbolic power to identify struggles over legitimate environmental conduct. Comparative reading across these bodies of work revealed three recurrent mechanisms—insulation, externalisation, and symbolic authority—that operate at different scales but can be connected through the distribution and conversion of capital. These dimensions were retained because together they link material protection, relational cost shifting, and classificatory power without assuming that they always coincide. The empirical domains discussed below serve as analytical illustrations of this synthesis, not as a representative sample or an independent empirical test.

The article first reconsiders the relationship between the environmentalism of the poor and the non-ecology of the rich. It then develops the three constitutive dimensions of the concept and explores their analytical purchase through existing research on mobility, environmentally privileged urban space, and private adaptation. These domains are not presented as original empirical evidence or as a systematic test; they specify how the framework can organise comparative inquiry. The final section considers the political consequences of shifting attention from individualised responsibility toward unequal relations of exposure, protection, externalization, and classificatory power.

The terms used in the analysis are therefore not interchangeable. Affluence refers primarily to command over economic resources and, often, to high levels of consumption; privilege denotes a relational advantage in access to environmental goods, protection, mobility, or voice; and elite position refers to a capacity to influence institutions, agendas, or authoritative classifications. A high-income household may possess substantial insulation without significant institutional power, whereas a corporation, professional group, urban development coalition, or transnational policy network may shape sustainability standards beyond the consumption of its members. The title retains the expression elite power because the framework includes these institutional and classificatory capacities, but the relevant unit of analysis must be identified separately in each empirical application.

## 2 Environmentalism of the poor and the relational structure of ecological inequality

One of the most influential contributions to contemporary ecological thought is Joan Martínez-Alier’s notion of the environmentalism of the poor (Martínez-Alier, 2002; Robbins, 2012). By foregrounding struggles over land, water, forests, extraction, pollution, and livelihood among marginalised populations, this perspective challenged the assumption that environmentalism was primarily a concern of affluent societies or post-materialist middle classes. It demonstrated that ecological conflicts frequently emerge where environmental degradation threatens the material and social conditions of life.

This insight remains crucial, but it should not be transformed into a sociological opposition between two homogeneous groups. The environmentalism of the poor does not imply that dominated populations possess an intrinsically ecological disposition, any more than affluence necessarily produces indifference towards ecological degradation. Nor are necessity and choice mutually exclusive principles that can be mapped directly onto “poor” and “rich.” Ecological practices emerge from heterogeneous social positions, and these positions may contain contradictory dispositions, interests, and forms of environmental engagement.

What Martínez-Alier’s perspective makes visible, however, is a structural asymmetry that remains analytically decisive: social groups encounter ecological constraints under profoundly unequal conditions. For populations whose livelihoods, health, housing, or territorial continuity are directly threatened by extraction, toxicity, scarcity, displacement, or infrastructural abandonment, ecological degradation can become an immediate condition of social reproduction. Environmental struggle may therefore arise not from a distinctive environmental identity but from conflicts over the conditions necessary to sustain life.

A relational approach requires examining the other positions within the same socioecological relations. Environmental harms are not simply suffered unevenly; the capacities to avoid, displace, mitigate, and define them are also unequally distributed. Corporations, states, financial institutions, high-consuming groups, philanthropic organizations, and transnational environmental actors occupy different positions and possess different combinations of economic, cultural, political, and symbolic resources. They therefore participate differently not only in the production of ecological pressures, but also in the organisation of responses to them and in struggles over their legitimate interpretation.

A Bourdieusian perspective is useful here because it directs attention away from fixed social categories and toward relations between positions endowed with unequal forms and volumes of capital (Bourdieu, 1993; Bourdieu and Wacquant, 1992). Ecological conflicts can consequently be understood simultaneously as material struggles over resources, infrastructures, exposure, and protection, and as symbolic struggles over classification: over who can define an ecological problem, whose knowledge is recognised, which practices count as responsible, and which solutions acquire legitimacy.

From this perspective, the environmentalism of the poor and the non-ecology of the rich are neither symmetrical concepts nor empirical descriptions of everything poor and affluent populations do. They illuminate different positions and mechanisms within an unequal socioecological space. The former directs attention to ecological struggles that emerge where environmental degradation threatens the conditions of existence; the latter identifies a positional logic made possible by concentrations of capital that can provide relative insulation from particular ecological constraints, facilitate the displacement of ecological costs, and confer disproportionate authority over legitimate responses to crisis.

The qualifier relative is important. Insulation is never complete: wealth does not place actors outside ecological relations or render them invulnerable to climate change, pollution, disaster, or ecological breakdown. Nor does it imply that affluent actors cannot develop genuine ecological commitments. It refers instead to an unequally distributed capacity to mediate exposure through mobility, technology, markets, infrastructures, spatial relocation, and access to protection. Likewise, dominated populations are neither uniformly territorially dependent nor reducible to vulnerability. What matters analytically is not an absolute opposition between vulnerability and freedom, but the unequal distribution of the resources through which vulnerability can be negotiated.

The non-ecology of the rich extends the relational question opened by the environmentalism of the poor. If the latter asks how ecological politics emerges where degradation threatens the conditions of life, the former asks how concentrations of capital alter the terms on which constraints are encountered, costs displaced, and responses defined. Their relation is not a binary opposition between ecological necessity and choice, but an asymmetry of exposure, protection, externalisation, and classificatory power. This provides the basis for examining the non-ecology of the rich as a structural configuration of unequal capacities rather than a moral attribute of affluent individuals.

## 3 The non-ecology of the rich: a relational configuration of power

The preceding discussion has situated the non-ecology of the rich within an unequal socioecological space. The task now is to specify the mechanisms through which privileged positions can alter relations to ecological crisis. I organise the concept around three interrelated capacities: insulation from ecological constraints, externalization of ecological costs, and symbolic authority over legitimate sustainability.

These capacities derive from the unequal distribution, field-specific value, and convertibility of capital. Economic capital can provide access to technologies, infrastructures, mobility, protected environments, and private adaptation. Cultural capital facilitates the appropriation of environmental knowledge and legitimate repertoires of ecological practice. Social capital provides access to networks, experts, and institutions through which resources and influence circulate. Symbolic capital consists in the recognised authority that allows these advantages to be perceived as competence, responsibility, or leadership (Bourdieu, 1984, 1993, 1998). Conversion is not automatic: economic resources become insulation when they secure protection; cultural and social resources become ecological authority when recognised within institutions; and institutional recognition can make a situated practice appear generally legitimate.

The socioecological space invoked here is not itself a single autonomous field. It is a relational configuration connecting struggles in political, economic, scientific, urban-development, consumption, and environmental-governance fields across local, national, and transnational scales. Each field validates different resources and gatekeepers: purchasing power may dominate housing and insurance markets, professional expertise may carry weight in planning and standards, and political or corporate office may confer agenda-setting authority. Buchholz's account of global fields cautions against treating transnational arenas as national fields writ large, while Jong's configurational field analysis emphasises historically contingent and shifting connections among fields, actors, and scales (Buchholz, 2016; Jong, 2024). This configurational approach supports the use of socioecological space as a comparative map of relations rather than a claim that one global ecological field has already been constituted.

Insulation concerns the capacity to reduce, mediate, or defer exposure to ecological constraints. Externalisation concerns the capacity to displace ecological costs across populations, territories, and temporal horizons. Symbolic authority concerns the capacity to define legitimate forms of ecological conduct and to make particular responses to crisis appear universal, responsible, or necessary. Together, these mechanisms connect material privilege to the political and symbolic organisation of ecological inequality.

The three dimensions are analytically distinct mechanisms and need not appear with equal intensity. A single mechanism—insulation, externalization, or symbolic authority—should normally be analysed through the more specific literatures on environmental privilege, ecological unequal exchange, or symbolic power. The term non-ecology of the rich is most useful for configurations in which at least two mechanisms are empirically connected, and it has greatest explanatory force when all three reinforce one another. Economic capital may, for example, purchase cooling, insurance, or relocation, while these protections depend on arrangements that redistribute costs and are legitimated as innovative or sustainable. The sequence can also run differently: symbolic or institutional authority may shape policy and investment rules that enable subsequent insulation and externalization. The framework therefore proposes testable relations rather than a fixed causal chain and requires researchers to establish, rather than assume, the connections in each setting.

### 3.1 Insulation from ecological constraint

The first dimension of the non-ecology of the rich is insulation from ecological constraint. Insulation does not imply immunity from ecological crisis. No social position lies outside ecological relations, and wealth cannot eliminate exposure to climate change, pollution, resource depletion, or environmental disruption. It refers instead to an unequally distributed capacity to mediate, reduce, postpone, or spatially displace particular forms of ecological constraint. Empirically, insulation may be spatial, through access to less exposed locations; infrastructural, through cooling, energy, water, or protective systems; financial, through insurance and liquidity; legal, through rights, contracts, and regulatory influence; or temporal, through the capacity to defer disruption and recovery costs.

A Bourdieusian perspective makes it possible to understand this capacity as relational rather than as the outcome of individual choice. Economic capital provides access to housing, technologies, infrastructures, mobility, insurance, and private forms of adaptation that can reduce exposure to environmental risks. Cultural capital facilitates the identification and appropriation of these protective strategies, while social capital can provide access to networks, information, institutions, and opportunities through which environmental risks are anticipated and managed. Ecological vulnerability is therefore mediated by social position and by the volume and composition of resources actors can mobilise.

Insulation also changes the practical experience of ecological limits. Where resources are scarce, environmental constraints may narrow the range of available responses; where capital is abundant, the same constraints can more readily be encountered through substitution, technological mediation, mobility, spatial relocation, or private adaptation. The distinction is one of degrees and capacities rather than absolute conditions. Affluent actors remain vulnerable, and less privileged actors are not devoid of agency or adaptive resources. What is unequally distributed is the capacity to create distance between ecological disruption and its immediate consequences for everyday life.

Insulation thus identifies a first structural feature of ecological privilege: the capacity to negotiate ecological limits through resources that are themselves unequally distributed. It is precisely because this capacity is relative rather than absolute that it can operate across different scales and contexts. Its significance lies not in allowing affluent actors to escape ecology, but in enabling some of the constraints generated by ecological crisis to be selectively mediated, deferred, or avoided.

### 3.2 Externalization of ecological costs

The second dimension of the non-ecology of the rich is the externalisation of ecological costs. If insulation concerns the unequal capacity to create distance from ecological constraints, externalisation concerns the social and spatial relations through which the costs associated with particular ways of living, producing, consuming, and adapting are displaced onto other populations, territories, and temporal horizons. The two mechanisms are closely connected but analytically distinct: insulation concerns protection from constraint; externalisation concerns the redistribution of the burdens that make such protection and consumption possible.

Political ecology has long shown that environmental degradation is inseparable from unequal relations of production, appropriation, and exchange. Extraction, pollution, waste, land degradation, and infrastructural disruption are not randomly distributed across space. They are organised through political-economic relations that connect sites of consumption and accumulation to distant sites where environmental costs are concentrated (Martínez-Alier, 2002; Pellow, 2007; Moore, 2015). From this perspective, ecological inequality is relational: the environmental advantages enjoyed in one place may depend upon the concentration of ecological burdens elsewhere.

The concept of the non-ecology of the rich brings this insight into the analysis of privileged positions. Affluent lifestyles and protected environments do not simply have ecological “impacts”; they are sustained through socioecological relations that frequently separate the sites where benefits are enjoyed from those where costs are borne. High levels of mobility depend upon infrastructures, energy systems, and extractive chains whose environmental consequences extend far beyond the point of use. Green technologies similarly require materials, production processes, land, energy, and waste infrastructures distributed across unequal geographies. Protected residential environments depend upon flows of labour, resources, energy, and infrastructure that exceed their spatial boundaries. What appears locally as ecological improvement or protection may therefore coexist with the displacement of ecological pressures across space.

Externalization should not, however, be understood as the conscious strategy of every affluent individual. It may result from deliberate corporate or political decisions, but it is more commonly a systemic capacity embedded in provision, production, infrastructure, property, finance, and exchange. Actors can benefit from ecological externalization without intentionally seeking to transfer harm elsewhere. Nor should every environmental advantage be presumed to create a corresponding disadvantage: the relation between a benefit and displaced social or ecological costs must be demonstrated empirically. The analytical question is therefore not one of individual culpability, but of position and mechanism: who can appropriate environmental benefits while remaining relatively distant from the costs through which they are produced, and through which institutions is that separation organised?

This also distinguishes externalization from carbon inequality alone. Unequal emissions provide an essential measure of the disproportionate contribution of high-income groups to climate change (Chancel, 2022; Kenner, 2019), but the relational structure extends beyond the quantity of emissions attributable to particular groups. It concerns the capacity to organise ecological relations so that the benefits of resource-intensive practices are concentrated while significant portions of their environmental and social costs are spatially, socially, or temporally displaced.

Externalisation can therefore operate at multiple scales. Within cities, environmental amenities and protected spaces may coexist with the displacement of pollution, undesirable infrastructures, or vulnerable populations. At national and transnational scales, consumption and technological transition may rely upon extractive and productive processes located elsewhere. Temporally, present forms of protection and consumption may transfer ecological costs to future populations. These forms differ empirically and should not be treated as equivalent, but they share a relational principle: the separation between the appropriation of benefits and the bearing of ecological costs.

Seen in this way, externalisation is not simply a consequence of affluent consumption but a condition that helps reproduce relative insulation itself. The ability to inhabit cleaner, safer, or more resilient environments can depend upon ecological relations extending well beyond those environments. Insulation and externalisation thus form two sides of an unequal socioecological relation: some actors possess greater capacity to reduce their exposure precisely because ecological pressures can be redistributed through infrastructures, markets, territories, and time.

At the macro-structural level, externalization is organised through historically constituted core–periphery relations. Colonial and postcolonial divisions of labour have concentrated extraction, polluting production, waste disposal, and ecological disruption in territories whose populations possess less power to refuse them, while benefits accrue disproportionately to firms and consumers located elsewhere (Escobar, 1996; Pulido, 2000; Sassen, 2014; Moore, 2015). The non-ecology of the rich therefore has a geopolitical dimension: insulation in privileged regions may depend upon commodity chains, unequal exchange, and political arrangements that displace ecological pressures toward peripheral territories. The core–periphery distinction should not be treated as a fixed geography, however, because peripheralized populations and sacrifice zones also exist within affluent countries, while privileged enclaves are found within poorer ones.

These processes are also racialized and gendered. Colonial histories have shaped whose territories are treated as extractive frontiers, whose labour absorbs environmental and reproductive costs, and whose exposure is politically normalised. Race, gender, class, and citizenship do not simply add separate disadvantages to the framework; they help constitute the relations through which some actors gain protection and mobility while others bear displaced risks. Insulation and externalization must therefore be investigated as intersectional processes whose institutional forms vary across countries and historical configurations.

### 3.3 Symbolic authority and the definition of legitimate sustainability

The third dimension of the non-ecology of the rich is symbolic authority over the definition of legitimate sustainability. Ecological inequality concerns not only unequal exposure to environmental harm and unequal capacities to externalize its costs, but also unequal power to define how ecological problems are perceived, classified, and addressed. Sustainability is therefore a terrain of symbolic struggle: actors occupy unequal positions in determining which practices count as responsible, which forms of knowledge are recognised as authoritative, and which responses to ecological crisis appear realistic, desirable, or legitimate.

A Bourdieusian perspective is particularly useful for understanding this dimension. Symbolic power operates through the capacity to make particular classifications of the social world appear natural, universal, and self-evident rather than socially situated (Bourdieu, 1993). Within socioecological relations, this capacity is unequally distributed because the authority to speak legitimately about ecological crisis is itself connected to the possession and convertibility of different forms of capital. Economic resources can provide access to environmentally valued goods, technologies, infrastructures, institutions, and platforms. Cultural capital enables familiarity with legitimate environmental vocabularies, scientific knowledge, and recognised forms of ecological conduct. Social capital facilitates access to networks in which environmental agendas are formulated and circulated. When these resources are recognised as legitimate, they can be converted into symbolic capital and ecological authority.

This process helps explain how socially situated practices acquire the appearance of universal environmental norms. Conscious consumption, technological substitution, carbon compensation, energy-efficient housing, and low-carbon mobility may all generate environmental benefits, but they also form repertoires of conduct whose accessibility is socially differentiated. When such repertoires become normative benchmarks, the unequal resources required to perform them can disappear from view.

Here the concept of symbolic violence becomes relevant. Symbolic violence does not require deliberate manipulation or explicit coercion; it operates when historically and socially produced classifications are recognised as legitimate and thereby conceal the relations of power embedded within them (Bourdieu and Passeron, 1977). Applied to sustainability, this mechanism can transform differences in resources and infrastructures into apparent differences in ecological virtue. Practices enabled by capital can be recognised as evidence of responsibility, while practices constrained by income, housing, mobility systems, or existing infrastructures can be interpreted as failures of environmental commitment.

The conversion of capital is central to this process. Economic capital can purchase access to goods and infrastructures coded as sustainable; cultural capital provides competences through which ecological practices are selected, interpreted, and displayed; these advantages can in turn generate symbolic recognition. Sustainability thus becomes one medium through which existing resources are converted into ecological legitimacy. The argument concerns the unequal social conditions under which practices become visible, recognisable, and authoritative, not the sincerity of individual ecological commitments.

Symbolic authority also operates beyond individual lifestyles, but it should not be conflated with every form of power involved in sustainability governance. Cultural distinction, recognised expertise, and classificatory power become forms of symbolic authority insofar as actors and categories are acknowledged as legitimate. Agenda-setting capacity and formal regulatory authority are better treated as distinct forms of institutional power: they allocate attention, enact standards, and organise compliance. Yet institutional position can generate symbolic effects by authorising particular experts, metrics, and solutions, while accumulated symbolic authority can facilitate access to institutional decision-making. Corporations, foundations, experts, governmental institutions, international organizations, and environmental actors possess these capacities in different combinations. Carbon accounting, certifications, technological solutions, market mechanisms, and models of sustainable urban development are therefore not merely technical instruments; their authority also depends on institutional and epistemic resources.

These classificatory struggles are neither uncontested nor unidirectional. Environmental justice movements, Indigenous mobilisations, anti-extractivist struggles, labour organizations, and climate justice movements produce alternative ecological knowledges and challenge dominant definitions of sustainability. The socioecological space is therefore a field of contestation rather than a system of complete symbolic domination. What remains unequal is the capacity of different actors to make their definitions circulate as authoritative, technically credible, institutionally actionable, or universally applicable.

The non-ecology of the rich thus describes neither a coherent elite ideology nor a catalogue of environmentally damaging behaviours. It identifies a relational configuration in which concentrations of capital can shape simultaneously the experience, distribution, and legitimate representation of ecological limits. The three dimensions may vary in intensity and combination across contexts, but together they provide an analytical framework for examining how ecological inequality is reproduced through material, spatial, and symbolic relations.

## 4 Spatializing the non-ecology of the rich: analytical sites and existing evidence

The preceding sections have developed the non-ecology of the rich as a framework organised around insulation, externalization, and symbolic authority. The three domains examined here were selected as strategically contrasting sites at household, urban, and transnational scales where protection, displaced costs, and legitimation can be studied together: mobility links ecological pressure to the capacity for spatial exit; environmentally privileged urban space makes the territorial production of protection visible; and private adaptation reveals the marketisation of ecological security. They are analytical illustrations, not equivalent phenomena, a representative sample, or an independent empirical test. Other settings—including corporate sustainability strategies, extractive industries, and carbon-offset markets—may configure the mechanisms differently and provide important comparative tests. The aim is to specify what becomes visible when existing research on these domains is read through the combined lenses of the three mechanisms.

### 4.1 Mobility: high-carbon practices and legitimate movement

Mobility offers a particularly revealing site for examining this configuration because access to fast, flexible, and resource-intensive movement is both socially stratified and ecologically consequential. Research on carbon inequality has demonstrated that high-income groups contribute disproportionately to global emissions and that affluent consumption patterns play a major role in this distribution (Chancel, 2022; Kenner, 2019). Mobility is one important component of this inequality. Evidence on the highly concentrated distribution of aviation provides additional support for this analysis: a small minority of frequent flyers accounts for a disproportionate share of passenger air-travel emissions (Gössling and Humpe, 2020).

The analytical relevance of mobility to the non-ecology of the rich, however, does not lie only in the quantity of emissions produced by affluent travellers. It lies in the combination of capacities that unequal access to mobility can entail. Mobility itself can operate as a form of insulation, allowing actors to move between places, avoid particular environmental constraints, choose residential and leisure environments, or relocate temporarily or permanently in response to risk. The capacity to move is therefore not merely an ecological burden; it can also constitute a resource for negotiating ecological vulnerability.

At the same time, high levels of mobility depend upon infrastructures, energy systems, extraction, production, and waste chains whose ecological costs extend far beyond the point of use. In this sense, mobility illustrates externalisation: the benefits of movement can be concentrated among mobile populations while significant portions of its environmental costs are distributed across distant territories and populations.

The symbolic dimension complicates the picture further. Technological substitution, electric vehicles, carbon compensation, sustainable aviation initiatives, and other attempts to reduce the ecological impact of mobility may generate genuine environmental improvements. Yet they can also make it possible to frame continued high levels of mobility as compatible with ecological responsibility. The relevant question is therefore not whether these technologies are “really” green or merely forms of hypocrisy, but which forms and levels of mobility become normalised as compatible with legitimate sustainability, and who possesses the resources to access them.

Read through the non-ecology of the rich, mobility thus brings together impact, protection, displacement, and legitimacy. Income, passports, flexible work, and transport infrastructures enable insulation through movement; energy, mineral, labour, land, and waste chains distribute associated costs beyond the mobile user; and airlines, vehicle manufacturers, certification bodies, experts, and public authorities participate in classifying particular forms of mobility as sustainable. The case shows why carbon inequality cannot be understood only as unequal emissions: mobility is simultaneously an ecological pressure, a resource for negotiating vulnerability, and a field of institutional struggle over legitimate movement. Whether these relations converge in a given transport system remains an empirical question.

### 4.2 Environmental privilege and the production of protected urban space

Urban space provides a second analytical site. Research on environmental justice and green gentrification has demonstrated that environmental goods and harms are unevenly distributed and that environmental improvement itself can reproduce exclusion and socio-spatial inequality (Checker, 2011; Anguelovski, 2016; Gould and Lewis, 2017). The literature on environmental privilege similarly directs attention to the unequal capacity to access desirable environmental conditions and protection from ecological harm (Park and Pellow, 2011). Comparative research on climate gentrification likewise shows how green and resilience investments can increase displacement pressures for poor and vulnerable residents (Anguelovski et al., 2019).

These findings allow urban sustainability to be reconsidered through the concept of insulation. Access to energy-efficient housing, green infrastructure, thermal comfort, cleaner environments, resilient infrastructures, and less exposed locations can reduce the immediate consequences of ecological risk. Such protection is not absolute, but access to it is structured by income, property, infrastructure, and spatial position.

Protected urban environments also reveal the relational character of externalization. Environmentally desirable spaces remain connected to wider systems of labour, energy, resources, transport, production, and waste. Their environmental quality cannot therefore be analysed within their spatial boundaries alone. The production of localised ecological advantage may coexist with environmental pressures distributed elsewhere in the urban region or along longer infrastructural and commodity chains.

Finally, environmentally privileged spaces can acquire symbolic significance as models of desirable urban futures. Green districts, energy-efficient developments, and resilient neighbourhoods may become showcases of ecological modernization. Again, the argument is not that such interventions lack environmental value. It is that their presentation as generalizable models can obscure the unequal resources required to access them and the wider socio-spatial relations upon which they depend.

The non-ecology of the rich therefore does not replace environmental privilege or green gentrification. It asks how the spatial advantages identified by these literatures intersect with externalisation and symbolic authority. Property ownership, income, credit, and planning access can secure insulation in greener and more resilient districts; displacement, rising rents, construction impacts, and regional resource flows may distribute costs beyond their beneficiaries; and municipalities, developers, planners, investors, and rating systems can authorise such districts as models of sustainable urbanism. These connections cannot be inferred from greening alone. They must be traced through the coalitions, infrastructures, and distributional effects of particular projects.

### 4.3 Private adaptation and insulated futures

A third analytical site concerns the increasing role of private resources in adaptation to ecological instability. Insurance, housing technologies, private energy systems, climate-resilient developments, cooling infrastructures, and other market-mediated forms of protection raise a fundamental question for political ecology: to what extent can adaptation itself become socially stratified? Research on the privatisation of climate adaptation shows how market-based insurance can shift responsibility for disaster recovery towards individuals and weaken solidaristic forms of collective protection (Lucas and Booth, 2020). More broadly, adaptation interventions can reproduce or create vulnerability when they overlook unequal participation, political framing, and the multi-scalar drivers of risk (Eriksen et al., 2021).

The relevance of these practices to the non-ecology of the rich lies first in insulation. Where protection from heat, flooding, energy disruption, water scarcity, or other environmental risks can be purchased privately, economic capital expands the range of adaptive responses available to particular actors. Ecological vulnerability is not eliminated, but its immediate consequences can be mediated through resources unavailable to others.

Private adaptation also raises questions of externalization. Protective infrastructures consume resources, energy, land, labour, and materials, and their ecological consequences may extend beyond the populations they protect. More importantly, private exit from collective systems can redistribute costs. Evidence from Cape Town, for example, shows that wealthier households responded to drought by substituting privately financed groundwater for public water, reducing utility revenues and shifting part of the burden toward poorer households (Abajian et al., 2026). The expansion of private solutions can therefore alter the political organization of adaptation when protection becomes a differentiated good rather than a collectively provided infrastructure.

The symbolic stakes are equally significant. Privately financed resilience may be represented as innovation, preparedness, or exemplary adaptation. Insurers, developers, technology firms, consultants, utilities, and public agencies can give these classifications institutional force through pricing, standards, investment decisions, and planning. Such arrangements may normalise a model in which ecological security is achieved through differentiated access to markets and technologies rather than collective provision and redistribution. The analytical issue is not private adaptation in itself, but whether purchased protection relies on displaced costs and how specific actors acquire authority to present it as a generally desirable response.

These examples show why the non-ecology of the rich is best treated as a conceptual framework rather than a description of a single empirical population. Mobility, protected urban space, and private adaptation involve different actors, institutions, scales, and causal mechanisms. The framework poses a common set of relational questions across them: Who can create distance from ecological constraints? Where are the costs of that protection displaced? Who can define particular responses as legitimate sustainability? It does not assume that the three mechanisms always occur together or operate identically; their combinations and causal relations remain questions for empirical investigation.

## 5 Political implications: from individual responsibility to socioecological power

The concept of the non-ecology of the rich has implications for how ecological responsibility is politically framed. If exposure to ecological constraints, the capacity to displace their costs, and the authority to define legitimate responses are unequally distributed, sustainability cannot be understood solely as a problem of individual conduct. It must also be analysed as a question of socioecological power.

Prominent consumer-oriented strands of sustainability discourse address individuals as formally equivalent ecological subjects. Consumers are encouraged to reduce waste, modify diets, calculate carbon footprints, adopt low-carbon technologies, and make environmentally responsible choices. Such practices may produce meaningful environmental benefits, and individual action is not politically irrelevant. Moreover, sustainability governance also includes regulatory, infrastructural, corporate, municipal, redistributive, and environmental-justice approaches that cannot be reduced to consumer responsibility. The problem identified here arises specifically when differences in ecological conduct are detached from the unequal infrastructures and resources that structure the capacity to act.

From a Bourdieusian perspective, this detachment produces a form of misrecognition. Practices enabled by economic and cultural capital can appear as the expression of individual ecological virtue, while practices constrained by income, housing, transport systems, employment, or access to infrastructures can be interpreted as failures of responsibility. Structural differences in the capacity to act are thereby translated into moral differences between actors.

The individualization of responsibility also narrows the political field within which ecological crisis is understood. Consumption practices are socially organised through broader systems of provision rather than generated by isolated individual preferences (Welch and Warde, 2015). Fossil-fuel infrastructures, industrial production, global transport networks, patterns of urban development, extractive systems, and institutional investment decisions structure both ecological impacts and the alternatives available to individuals. An exclusive emphasis on personal conduct can therefore obscure the institutions and relations through which environmentally consequential choices are produced.

Sustainability discourse is heterogeneous and contested, and it has enabled important political transformations. Its depoliticizing effect emerges when ecological responsibility is detached from unequal relations of exposure, protection, externalisation, and power, so that structurally differentiated capacities appear as universally available choices.

Technocratic forms of ecological governance can reinforce this displacement. Carbon accounting, environmental indicators, certifications, ESG frameworks, and other forms of expertise make ecological processes visible and governable in important ways. Yet metrics also classify: they establish what is measured, which impacts count, what constitutes improvement, and which interventions are considered feasible. Expertise is therefore not external to socioecological struggles over definition and legitimacy. As Latour (2017) emphasises, ecological crisis concerns political conflicts over territories, resources, infrastructures, and forms of collective life, not merely technical problems awaiting optimised management.

The non-ecology of the rich redirects attention from the morality of individual lifestyles towards these relations of power. The central political question is not whether affluent individuals are sincere environmentalists or hypocritical consumers. Nor is it whether less affluent populations make sufficiently sustainable choices. It is how socioecological arrangements distribute the capacities to produce environmental pressures, avoid their consequences, transfer their costs, access protection, and define legitimate solutions.

This shift also changes the meaning of ecological limits. Limits are not experienced in socially equivalent ways. For some actors, ecological constraints may appear primarily as restrictions to be managed through technological substitution, markets, mobility, or private adaptation; for others, they may take the form of exposure, scarcity, displacement, deteriorating infrastructures, or threats to livelihood. A political ecology of limits must therefore ask not only where ecological boundaries lie, but who is required to live with particular limits, who possesses the resources to negotiate them, and how the costs of doing so are distributed.

This is where the environmentalism of the poor and the non-ecology of the rich can be brought back into relation without reducing them to opposing social identities. Environmental justice, Indigenous, anti-extractivist, labour, and climate justice struggles challenge not only environmental harms but also the unequal distribution of authority over ecological futures. They make visible alternative claims about whose knowledge counts, how resources should be governed, where ecological costs should fall, and which forms of life should be protected.

The political implication of the argument is therefore a shift from sustainability as individualised virtue to sustainability as a problem of distribution, infrastructure, and democratic power. Ecological transition cannot be evaluated only by the diffusion of green practices or technologies. It must also be assessed according to how it redistributes exposure and protection, whether it reduces or reproduces externalization, and whose definitions of a viable ecological future acquire institutional force.

## 6 Conclusion

This article has developed the non-ecology of the rich as a concept for analysing a structurally privileged relation to ecological crisis. Building on research on carbon inequality, environmental privilege, ecological habitus, consumption, environmental gentrification, and green capitalism, it connects three dimensions of socioecological power: insulation from ecological constraints, externalization of ecological costs, and symbolic authority over legitimate sustainability. Its contribution to the sociology of environmental stratification is to analyse these dimensions together as unequal capacities to negotiate, redistribute, and define ecological limits.

This relational formulation also qualifies the contrast with the environmentalism of the poor. Neither concept names a homogeneous social group or fixed disposition. Martínez-Alier foregrounds conflicts that emerge where degradation threatens the conditions of life; the non-ecology of the rich foregrounds the resources through which constraints can be negotiated, costs displaced, and responses defined. The relation is one of structural asymmetry rather than categorical opposition.

Mobility, environmentally privileged urban spaces, and private adaptation illustrate how ecological inequality can combine differentiated protection, displaced costs, and struggles over legitimacy. The framework does not presume that every advantage produces harm elsewhere or that all three mechanisms always reinforce one another. It directs empirical research to establish the actors, institutions, scales, and conversion processes through which they are connected, and to identify cases in which these connections are absent, contested, or interrupted.

The concept therefore offers a focused research agenda rather than a totalizing description of elite environmental behaviour. Which forms of capital enable protection from ecological constraints, and for which households, organizations, or territories? Through which supply chains, infrastructures, labour relations, and temporal arrangements are costs displaced? Which experts and organizations can classify responses as legitimate sustainability? Under what conditions is material privilege converted into symbolic ecological authority, and when do the three mechanisms reinforce one another? Finally, which public institutions, collective infrastructures, regulations, and justice movements can interrupt these conversions? Comparative research around these questions can specify the scope conditions and causal sequences of the framework.

For political ecology, this perspective has a broader implication. Ecological transition cannot be evaluated solely through aggregate reductions in impact or the diffusion of sustainable technologies. It must also be assessed by how it redistributes exposure, protection, costs, and authority. A transition that expands environmental protection while concentrating access to it, or reduces impacts in one place by transferring them elsewhere, may reproduce socioecological inequality. Ecological limits are not simply biophysical thresholds confronting an undifferentiated humanity; they are encountered through unequal social relations and infrastructures.

The three dimensions can be operationalized through complementary qualitative and quantitative strategies. Observed inequalities are indicators or outcomes, not in themselves evidence of the mechanisms that produced them. Insurance coverage or access to resilient infrastructure, for example, measures the distribution of protection; identifying insulation requires tracing how income, property, networks, regulation, and institutional access are converted into reduced, mediated, or deferred exposure. Comparative cases, process tracing, and ethnography can therefore distinguish an unequal outcome from the social relations generating it. Externalization can be traced through material-flow and emissions data, supply and waste chains, the spatial incidence of environmental harm, and multi-sited studies following how benefits and costs are separated across populations and territories. Symbolic authority can be examined through discourse, document, field, and network analysis of policy processes, expert bodies, sustainability standards, certifications, philanthropic programmes, and media visibility. Relevant indicators include who sets evaluative categories, whose expertise is institutionally recognised, which definitions circulate across arenas, and which proposed solutions become technically credible and politically actionable. These indicators do not turn the framework into a fixed index; they provide an empirical research programme for comparing how the mechanisms combine across settings and scales.
